# Detection of epileptic activity in presumably normal EEG

**DOI:** 10.1093/braincomms/fcaa104

**Published:** 2020-09-23

**Authors:** Sara Baldini, Francesca Pittau, Gwenael Birot, Vincent Rochas, Miralena I Tomescu, Serge Vulliémoz, Margitta Seeck

**Affiliations:** f1 EEG and Epilepsy Unit, Neurology Department, University Hospitals and Faculty of Medicine of Geneva, Geneva, Switzerland; f2 Functional Brain Mapping Laboratory, Department of Fundamental Neurosciences, University of Geneva, Geneva, Switzerland

**Keywords:** interictal spikes, scalp voltage maps, pharmacoresistant epilepsy, EEG

## Abstract

Monitoring epileptic activity in the absence of interictal discharges is a major need given the well-established lack of reliability of patients’ reports of their seizures. Up to now, there are no other tools than reviewing the seizure diary; however, seizures may not be remembered or dismissed voluntarily. In the present study, we set out to determine if EEG voltage maps of epileptogenic activity in individual patients can help to identify disease activity, even if their scalp EEG appears normal. Twenty-five patients with pharmacoresistant focal epilepsy were included. For each patient, 6 min of EEG with spikes (yes-spike) and without visually detectable epileptogenic discharges (no-spike) were selected from long-term monitoring recordings (EEG 31–37 channels). For each patient, we identified typical discharges, calculated their average and the corresponding scalp voltage map (‘spike-map’). We then fitted the spike-map for each patient on their (i) EEG epochs with visible spikes, (ii) epochs without any visible spike and (iii) EEGs of 48 controls. The global explained variance was used to estimate the presence of the spike-maps. The individual spike-map occurred more often in the spike-free EEGs of patients compared to EEGs of healthy controls (*P* = 0.001). Not surprisingly, this difference was higher if the EEGs contained spikes (*P* < 0.001). In patients, spike-maps were more frequent per second (*P* < 0.001) but with a shorter mean duration (*P* < 0.001) than in controls, for both no-spike and yes-spike EEGs. The amount of spike-maps was unrelated to clinical variables, like epilepsy severity, drug load or vigilance state. Voltage maps of spike activity are present very frequently in the scalp EEG of patients, even in presumably normal EEG. We conclude that spike-maps are a robust and potentially powerful marker to monitor subtle epileptogenic activity.

## Teaser

This study aimed to detect epileptic activity in presumably normal EEG by scalp voltage map of interictal epileptic discharges. This ‘spike-map’ was quantified in EEGs with or without spikes. The high presence in EEG without detectable epileptic activity suggested that ‘spike-map’ could serve as a marker to monitor subtle epileptogenic activity.

## Graphical Abstract

**Graphical Abstract:**
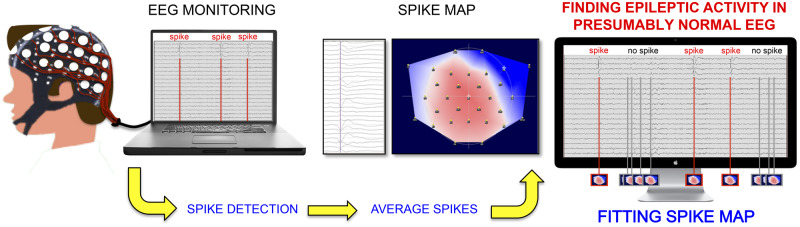

## Introduction

Epilepsy is one of the most frequent chronic neurological disorders, with a prevalence of 0.5–1% in the general population (Sander, 2003). A challenging problem in managing patients is to determine if their seizures are, in fact, controlled by the prescribed medical treatment given the unreliability of patient self-report and the absence of devices that reliably monitor seizure frequency (Cook *et al.*, 2013). To close this gap, an objective marker of ongoing epileptogenic activity is needed. Ideally, such a marker would be based on EEG well established that the detection of interictal epileptic discharges in scalp EEG (comprising spike-waves, polyspike-waves or spikes; hereunder called spikes) has a low sensitivity. Even in patients with very active epilepsy, a 20–30 min standard EEG (as it is usually practiced), may contain no spikes and thus be incorrectly reported as normal, i.e., making it an imperfect marker of active epileptogenicity in the individual patient. In clinical practice, EEG spikes are defined as graph elements with specific characteristics and morphological features, usually based on visual analysis of EEG curves (Gibbs *et al.*, 2002). However, in addition to this conventional approach, spikes can be also characterized by whole-scalp topographic maps specific to the epileptogenic focus localization (Scherg and Ebersole, 1993; Koenig *et al.*, 2002). Voltage maps have been proposed as a possible biomarker of different neuropsychiatric diseases (Ebersole and Wade, 1990; Lopes da Silva, 1990). Indeed, spike-specific voltage maps seem to be present even if no-spike-graphic element is visible in the EEG curves. In a combined EEG-functional magnetic resonance imaging study using caps with 32–94 electrodes, Grouiller *et al.* (2011) showed that specific voltage maps of epileptogenic discharges—‘spike-maps’—have haemodynamic correlates that are concordant with the epileptic focus (Grouiller *et al.*, 2011). They used long-term EEG recording results of patients with focal epilepsy to define the topographic voltage map of the spikes and then fitted the patient’s map to the ongoing EEG recorded in the scanner (without any visually detectable spikes). The time course of the spike-map correlated with the hemodynamic response function and showed blood oxygenation level-dependent changes in the epileptic zone, as validated by the post-operative result. This study strongly suggests the presence of spike-specific maps in the scalp EEG even in the absence of visible spikes, which could be a marker of ongoing activity in the epileptogenic focus area.

In the present study, we investigated whether spike-maps, obtained in a clinical setting (long-term video-EEG recording), can be detected in the EEG of patients with known active uncontrolled focal epilepsy but who showed no visible spikes during the EEG epochs. We were interested in evaluating the potential of the individual spike-maps as a marker to reveal visually undetected epileptic activity, given the low clinical yield of visual search for discharges in scalp EEG. To this end, we compared the frequency of spike-maps during periods of visually ‘normal’ EEG epochs versus epochs containing visible epileptogenic discharges in the same patient and to EEG epochs of a healthy control group.

## Materials and methods

### Subjects and recordings

We retrospectively analysed 25 patients with pharmacoresistant focal epilepsy who had been hospitalized for presurgical evaluation in 2013–15 at our Epilepsy Unit at the University Hospitals of Geneva. Inclusion criteria were: focal epilepsy, unifocal interictal epileptiform discharges (IEDs), artefact-free EEG obtained with at least 31 electrodes, >24 h since the last seizure. Exclusion criteria were: multifocal or generalized epilepsy, multifocal interictal discharges. Spikes were defined as transient events, distinguishable from the background activity with at least 2× higher amplitude than the background or with an evocative morphology, including a peak at conventional screen display and duration of 20–70 ms. A similar definition was used for sharp waves, but the duration could be up to 200 ms. For each patient, we selected two periods of 6 min, i.e., EEG with spikes (yes-spike-condition) and without any detectable spike during wakeful rest (no-spike-condition). The following electrodes were placed according to the 10–10 system: Fp1, Fp2, F7, F8, F3, F4, Fz, T3, T4, C3, Cz, C4, T5, T6, P3, Pz, P4, O1, O2, FC1, FC2, FC5, FC6, CP1, CP2, CP5, CP6, TP9, TP10, F9/10, T9/10 and P9/10; ref: FCz. Impedance was kept at <5 kΩ. EEG was recorded with a sampling rate at 256 Hz. Six minutes of wakeful resting-state EEG from 48 healthy subjects [24 female, age (mean ± SD) 32.9 ± 9.08 years] were also obtained and served as controls.

### EEG data pre-processing

The EEG was band-pass filtered offline between 1 and 40 Hz. Independent component analysis was applied to remove cardiac and oculomotor artefacts, based on the time course, the topography, and the waveform of the independent component analysis component (Jung *et al.*, 2000). Electrodes affected by artefacts were interpolated using a 3D spherical spline (Perrin *et al.*, 1989); the data were downsampled to 128 Hz and then recomputed to common average-reference. Voltage maps were computed at every sampling point. When the EEG-values at each sampling point and for each electrode of each electrode and time point are plotted across the scalp, a voltage map emerges. In order to visualize voltage maps, positive and negative values are computed and colour-coded. As established in the literature, positive values are red, negative amplitude values are blue and 0-values are white. Voltage maps are relatively robust regarding the amplitude of the signal, i.e., a large spike or subtle invisible spike lead to the same voltage map.

**Figure 1:**
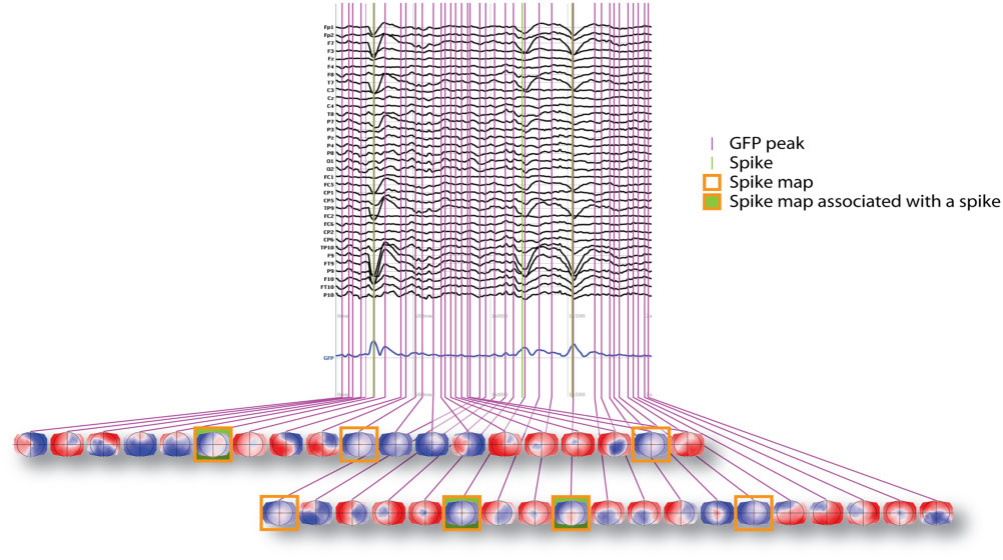
**Graphic illustration of spikes and microstate distribution in the EEG.** On the upper part, example of a 35-channels EEG (electrode labels on the left). Blue line: Global field potential (GFP) values corresponding to the time points of the EEG (sampling rate 256 Hz, downsampled to 128 Hz). Vertical purple lines indicate GFP maxima associated with a variety of maps, including the spike-map (voltage map of the epileptogenic interictal discharge). GFPs of the spike-map are framed by an orange box. Association of the spike-map to a visible spike or sharp wave is indicated by green filling of the orange box. Please note that spike-maps occur even without visible spikes.

In order to improve the signal-to-noise ratio, only the data at the time points of the local maxima of the global field power (GFP) were submitted to further analysis (Britz *et al.*, 2010). The GFP is a scalar measure of the strength of the scalp potential field and is calculated as the standard deviation of all electrodes at a given time point (Michel *et al.*, 1993; Lehmann *et al.*, 1998; Brunet *et al.*, 2011). Thus, GFP maxima reflect maximal synchronized neuronal activity (Skrandies, 1990). Voltage maps tend to be stable around the maxima, but do not carry any information on normal or pathological content. Any EEG can be described by a sequence of GFP maxima, i.e., voltage maps representing physiological brain activity like resting states as well as epileptogenic activity (spike-map). For the purpose of the study, we concentrate our analysis on the spike-maps (Fig. 1, orange boxes).

### Analysis

For each patient, we determined the individual spike-specific EEG voltage map using the averaged spikes typical for that patient. A board-certified EEG-expert (F.P., S.V., M.S.) marked the peak of an average of 15 spikes from the 6-min segment of the EEG with spikes. After band-pass filtering (1–40 Hz), the spikes were averaged aligned to the peak and the average EEG voltage map at the peak of GFP was considered as the ‘spike-map’ (Grouiller *et al.*, 2011). We fitted the spike-map on the GFP local maxima of (i) EEG of a patient with visible spikes (yes-spike-condition), (ii) EEG of the same patient without any visible spike (no-spike-condition) and (iii) EEGs of the 48 controls (Fig. 1). The spatial correlation between the spike-map and the recorded EEG at each time point of the two EEG-segments was computed (Michel and Murray, 2012). The presence of the epileptic map on such EEGs was quantified by the global explained variance (GEV) (Murray *et al.*, 2008). The GEV is the temporal mean of the absolute value of the spatial correlation weighted by the GFP. We also computed, among the GFP peaks of the entire EEG, the frequency of occurrence (how many times a voltage map is recurring per second) and mean duration (averaged amount of time, in ms, that a voltage map was continuously present) of the spike-map.

The EEG data pre-processing and analyses were performed with the freely available Cartool software package (release 3.51) (Brunet *et al.*, 2011).

### Statistical analysis

We assessed whether GEV, frequency and mean duration obtained from the patient’s EEG, with or without a spike, were statistically different from those of the control group composed of the 48 individuals. We performed the normality test (one-sample Kolmogorov–Smirnov test; *α* = 0.05) on the variables distribution for patients and controls, which confirmed normal distributions in all fitting combinations for each epileptic map. We then standardized GEV, frequency and mean duration for each patient by calculating the *Z*-score, i.e., subtracting the mean values of the controls from the patient’s value and then dividing the difference by the standard deviation of the controls. We thereby obtained standardized values of the GEV (sGEV), frequency and mean duration for each patient allowing comparison across all patients. This standardization allowed for statistical testing at the group level by comparing the variables distributions of the patient group (*N* = 25) with the reference distribution of the control group. The *P*-value is given by 1 − (*Z*−), where standard normal cumulative distribution function is the cumulative distribution function of the standard normal distribution.

The Pearson’s correlation coefficient was computed in order to assess whether GEV values, obtained in the EEG without spikes, were correlated to the following clinical variables: disease duration, age of onset and frequency of seizures per week. In addition, independent samples *t*-tests were performed to compare the GEV values between the following nominal variables: (i) lack of contact during seizure or not; (ii) presence or absence of psychiatric impairments; (iii) presence or absence of a structural lesion; (iv) presence or absence of tonic-clonic seizures and (v) number of antiepileptic drugs at the moment of hospitalization.

Even though the EEG was recorded during wakefulness, subtle background slowing indicative of light sleep was not excluded. Since spikes, and maybe also spike-maps, tend to occur more often during light sleep, we filtered each EEG in the low (1–7 Hz) and in the high (8–40 Hz) frequency band and performed the fitting of the individual spike-maps, separately, in these two filtered files. We compared the GEV values, respectively, for the no-spike and yes-spike condition, by a paired samples *t*-test.

Finally, we set out to determine if spike-maps were associated with subtle epileptogenic discharges, which escaped visual analysis. Topographic correlation between the spike-map and the map of each time frame of the raw no-spike-condition EEG was realized with the freely available Cartool software package (release 3.51) (Brunet *et al.*, 2011). Only correlations *r* ≥ 0.9 were considered ‘high’ and taken for the analysis. We selected and averaged the EEG from –500 ms to +500 ms around the time frame of spike-map (i.e. EEG highly suggestive of the presence of spike-map) to look for the presence of subtle spikes.

Statistical analyses were run on SPSS (version 20, IBM Corporation, Armonk, NY, USA). A value of *P* < 0.05 as considered statistically significant.

### Data availability

Raw data were gathered at the University Hospitals of Geneva, Epilepsy Unit. Derived data supporting the findings of this study are available from the corresponding author on request.

## Results

The clinical characteristics of the 25 patients are summarized in Table 1 [11 females, age (mean ± SD) 31 ± 3 years; range 11–58 years]. Average disease duration was 17 ± 3 years (mean ± SD; range 1–49 years). Magnetic resonance imaging (MRI) findings were heterogeneous; most patients had normal MRI or focal cortical dysplasia. Localization of epilepsy was noted in the temporal lobe in 11 of 25 patients, the remaining patients suffered from unifocal extra-temporal epilepsy (Table 1).

**Table 1:** Clinical characteristics of the patient cohort and antiepileptic drug treatment on admission

Patients	Age at evaluation/ sex/age at onset	Duration of disease	MRI	Epilepsy localization	Generalized tonic-clonic seizures	Average seizure frequency	Psychiatric comorbidity	AED1	AED2	AED3	AED4
1	43/F/35	8	N	RT	Yes	1/day	Anxiety	LTG 300	ZNS 50	VPA 1000	
2	9/M/1	8	N	LC	Yes	2/weeks	No	LTG 200	VPA 600	TPM 25	
3	26/F/13	13	N	LT	No	1/3 weeks	No	LTG 600	LEV 2000		
4	41/M/33	8	N	RT	Yes	1/month	No	VPA2000	CBZ 400		
5	16/M/8	8	N	RF	Yes	1x/year	No	LTG 400	LEV 2000		
6	38/M/24	14	N	LT	Yes	4/month	No	LCS 400	CBZ 400		
7	52/F/22	30	N	RT	No	5/week	No	OXC 900	TPM150		
8	24/F/12	12	N	LT	Yes	1/month	No	VPA 1600			
9	26/M/18	8	HS	LT	Yes	1/week	No	LEV 1000	LTG 600		
10	51/M/16	35	HS	LT	Yes	10/month	PNES	VPA 1600			
11	42/F/17	25	HS	LT	Yes	1/week	Depression	LTG 400	PRM 750		
12	20/F/9	11	FCD	ROF	No	5/day	No	CBZ 800	CLB 20	LTG 400	
13	41/M/24	17	FCD	RT	No	15/month	No	VPA 1000	LTG150	CBZ 1200	PER 10
14	31/F/7	24	FCD	LF	No	2/day	No	CLB 30	RFN 2400	LEV 2000	PB 300
15	55/F/6	49	FCD	RF	No	3/day	No	CBZ 400	LEV 2000		
16	35/M/25	10	FCD	LF	Yes	2/week	Narcisstic personality traits	CBZ 1200	VPA 1000		
17	12/F/1	11	FCD	LC	No	3/week	No	OXC 1050	VPA750	CNZ 0.5	
18	23/M/12	11	FCD	RT	Yes	8/year	No	TPM 300	LTG 300	LEV 2000	
19	18/F/11	7	FCD	LPO	Yes	2/day	No	CBZ 10000	ZNS 300		
20	50/M/1	49	FCD	LF	Yes	3/week	Depression	LTG 200	LEV 2000	CLB 10	LCS 400
21	13/M/8	5	FCD	RF	Yes	5/day	No	CBZ 500	LTG 100		
22	15/F/1	14	N	LPO	Yes	1/week	No	LTG 200	VPA 1800	CBZ 800	
23	15/F/13	2	DNET	LT	No	3/day	No	LTG 100	LEV 1000		
24	15/M/9	6	Haemorrhagic lesion	LCP	Yes	3/month	No	LTG 175	LEV 1000	VPA 750	
25	40/M/2	38	Post-traumatic lesion	RF	Yes	9/year	Depression, anxiety	CBZ 1200	CNZ 6		

CBZ = carbamazepine; CLB = clobazam; CNZ = clonazepam; DNET = dysembryoplastic neuroepithelial tumour; FCD = focal cortical dysplasia; HS = hippocampus sclerosis; LC = left-central; LCP = left-central-parietal; LCS = lacosamide; LEV = levetiracetam; LF = left-frontal; LPO = left-parietal-occipital; LT = left-temporal; LTG = lamotrigine; N = normal; OXC = oxcarbazepine; PNES = psychogenic non-epileptic epileptic seizure; PRM = primidone; PER = perampanel; PB = phenobarbital; RF = right-frontal; RFN = rufinamide; ROF = right-occipital-frontal; RT = right-temporal; TPM = topiramate; VPA = valproate; ZNS = zonisamide. All number refers to dosages in milligram.

**Figure 2:**
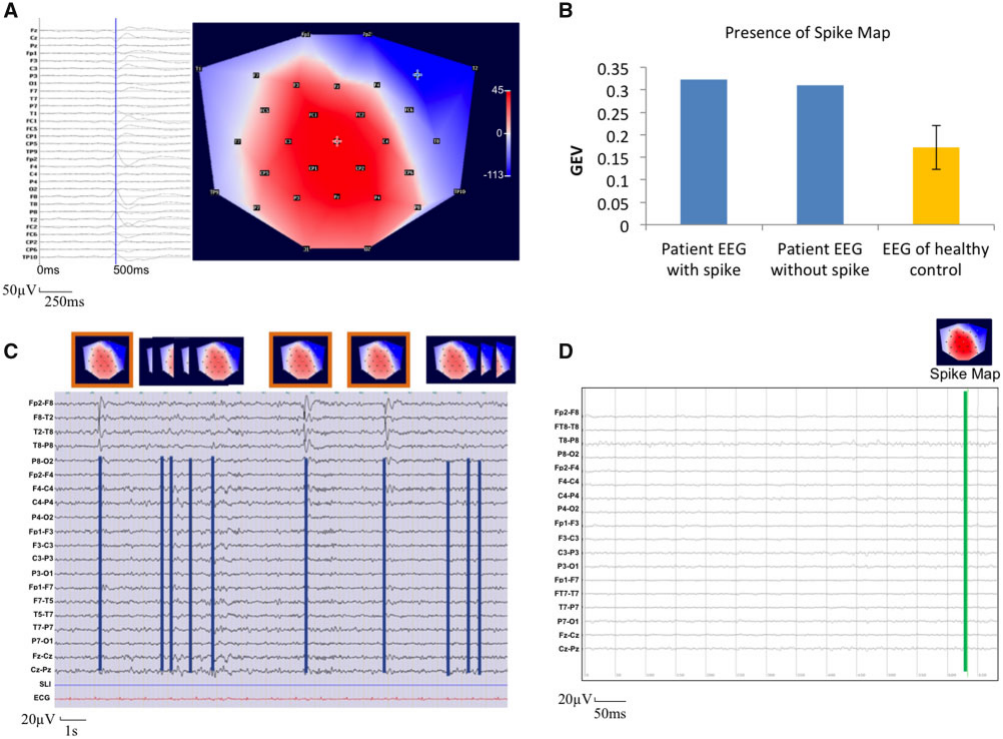
**An example of the procedure to detect the epileptic activity in presumably normal EEG by spike-map.** Patient no. 12 (right-frontal focus; F8). (**A**) Typical interictal epileptogenic discharge and corresponding spike-map (the bar on the right indicates, respectively, in blue the negative, in red the positive values of voltage expressed in µV). (**B**) Histogram regarding the presence of the spike-map in the 6-min EEGs of patient no. 12 with visible spikes (left) and presumably normal EEG (middle) compared to the control group (yellow). Whiskers: 2× standard error. The spike-map is much more frequently found in the patient’s EEGs, both in the EEG with and without visible spikes. (**C**) An example of the EEG of patient no. 12, containing spikes. The corresponding spike-map is indicated with an orange box. Spike-maps without visible spikes were also retrieved in this EEG (without orange box), more frequent than spikes. (**D**) EEG of healthy control. Only one map resembling the patient’s spike-map was found.

**Figure 3:**
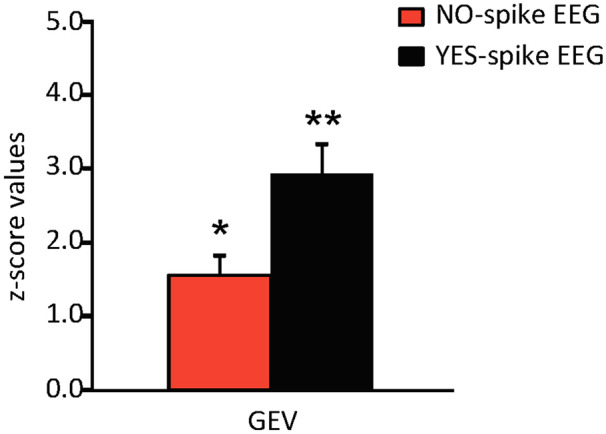
**Comparison of no-spike and yes-spike conditions of the EEG of all patients to healthy controls.** In both conditions, there was a major difference between the patient and control group. Compared to healthy controls (mean = 0 ± 1 SD), the EEG with spikes (**P < 0.001) and without spikes (*P = 0.001) contained significantly more spike-maps.

Statistical analysis using the standardized values revealed a significantly increased presence of the spike-map in the patient group compared to the controls (Figs 2 and 3). As expected, the difference was stronger for the EEGs with spikes (*t* = 7.035; df = 24; *P* < 0.001), but also highly significant for EEGs without spikes (*t* = 5.688; df = 24; *P* = 0.001). On an individual level, all but two patients differed clearly from the control group. In these two patients (Subjects 8 and 19) the GEV value of the epileptic map was higher in the EEG of controls than in the EEG without the spike of the patients. These two patients did not have any particular clinical characteristics in common, but the epileptic maps resembled one of the physiological maps, described by Britz *et al.* (2010) as ‘auditory microstate’ (Case 8) and ‘visual microstate’ (Case 19).

**Figure 4:**
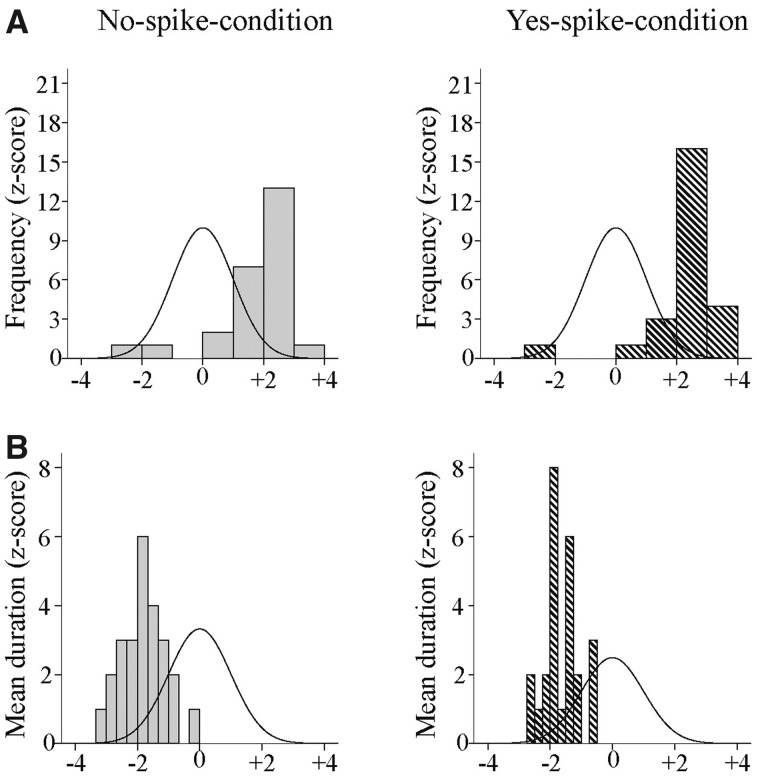
**Comparison between controls and the 25 patients using z-score normalization.** (**A**) The relative frequency of the spike-maps is higher in the EEG without spikes (left, *P* < 0.001) and with spikes (right, *P* = 0.001). The curve indicated the Gaussian distribution of the normative data (control group). (**B**) The mean duration of the spike-maps. They are significantly shorter, both in the EEGs without spikes (left, *P* < 0.001) and with spikes (right, *P* < 0.001) than in the control group.

We calculated the frequency of occurrence and the mean duration of the spike-maps in the patient group. Using normalization with z-score, we found that the patients had significantly more spike-maps compared to the controls (no-spike: *t* = 7.275, df = 24, *P* < 0.001; yes-spike: *t* = 9.148, df = 24, *P* < 0.001), but they were much shorter (no-spike: *t* = −12.860, df = 24, *P* < 0.001; yes-spike: *t* = −14.993, df = 24, *P* < 0.001) (Fig. 4).

GEV values of no-spike-condition EEGs were unrelated to disease duration (*r* = 0.303, *P* = 0.141), age of onset (*r* = −0.068, *P* = 0.746) or frequency of seizures per week (*r* = −0.169, *P* = 0.418). Moreover GEV was not different between temporal and extra-temporal lobe epilepsy (*t* = −1.494, df = 23, *P* = 0.149), presence or absence of a structural lesion (*t* = 1.455, df = 23, *P* = 0.159), presence or absence of psychiatric impairments (*t* = −0.018, df = 23, *P* = 0.986), or presence or absence of tonic-clonic seizure (*t* = 0.372, df = 23, *P* = 0.713). There was no correlation with the number of drugs being taken by the patient at the moment of hospitalization (*r* = 0.029; *P* = 0.269).

**Figure 5:**
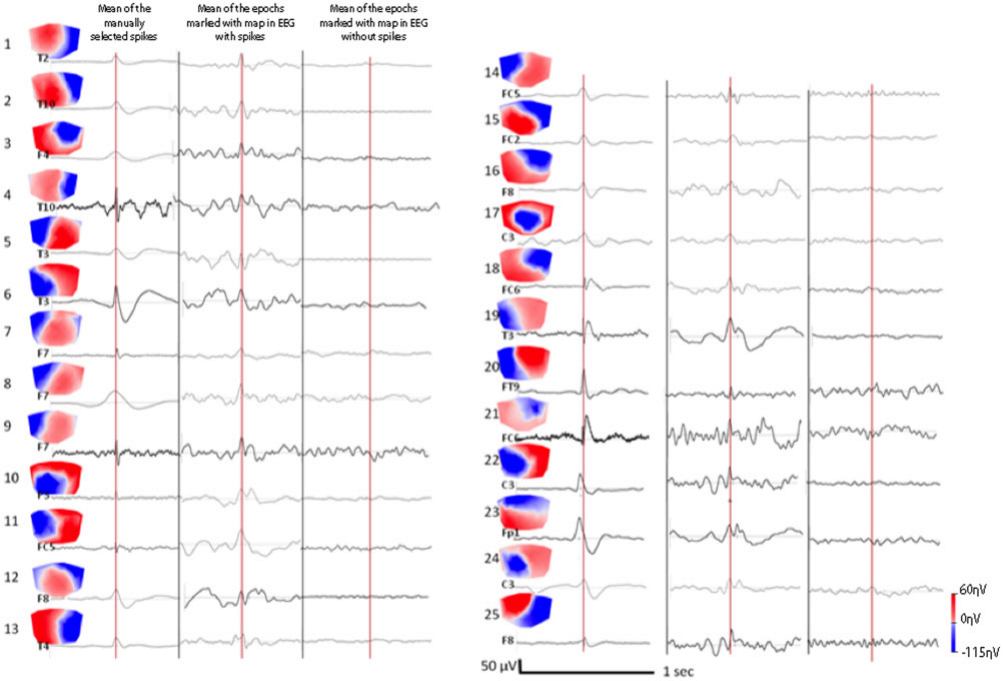
**No discharges visible even after averaging.** Left block: patients 1–13, right block: patient 14–25. First column (of each block): mean of the manually selected epileptogenic discharges with the correlated topographic epileptic map from the yes-spike-condition EEG. For each patient, a clear epileptogenic discharge appears in the average with a high signal-to-noise ratio. Second column: mean of the epochs around the time frame maximally correlated with the spike-map in the yes-spike-condition EEG, i.e., including spikes but also presumably normal EEG. Please note that the averaging procedure resulted in discharges of similar morphology as in the first column, but with a somewhat poorer signal-to-noise ratio. Third column: Same computation as in the second column for each patient but from the EEG without visible discharges (no-spike-condition). Average of the EEG epochs around the time frame of the spike-maps did not result in the detection of spikes, i.e., they did not correspond to small or missed spikes.

Analysis on the filtered EEG showed that the presence of the spike-map in patients compared to controls is independent of a specific frequency band (Table 2). Among patients, there were no differences between the 1–7 Hz band and the 8–40 Hz band (*t* = −0.485, df = 24, *P* = 0.632) in the EEG without visible spikes, suggesting that the presence of spike-map in the no-spike-condition is independent of the predominant background frequencies. In the EEG with visible spikes, the GEV of 8–40 Hz was significantly higher when compared to the 1–7 Hz filtered EEG (*t* = −3.506, df = 24, *P* < 0.001), due to the presence of spikes, which are by definition characterized by higher frequencies. In order to confirm that there was no discernible epileptogenic activity in the scalp EEG itself and verify that there were no overlooked small-amplitude visible spikes, we averaged the time frames at the moment of maximal correlation with the spike-map in the no-spike-condition condition. We did not see any graph elements resembling a spike in any of the patients (Fig. 5).

**Table 2:** Comparison between spike-maps selectively for low and high frequencies

EEG of patients (*n* = 25)	Frequency bands	GEV	
		*Z*-score ± SD	*P*-value
No-spike-condition	1–7 Hz versus controls	1.4 ± 2.2	0.001
	8–40 Hz versus controls	1.6 ± 1.9	0.001
Yes-spike-condition	1–7 Hz versus controls	2.3 ± 2.5	<0.001
	8–40 Hz versus controls	3.4 ± 2.7	<0.001

For both slow (1–7 Hz) and high (8–40 Hz) frequency bands, the spike-maps were significantly more frequent in the patient population, suggesting that spike-maps are independent of vigilance states. No-spike-condition = EEG without epileptogenic discharges; yes-spike-condition = EEG containing epileptogenic discharges.

## Discussion

To the best of our knowledge, this is the first study exploring the use of epileptogenic discharge-related voltage topographic maps (‘spike-maps’), computed from scalp EEG, as a marker of epileptogenic activity in patients with known focal epilepsy. These maps were found significantly more often in patients compared to controls, independent of the presence of visible epileptogenic discharges. Spike-maps therefore appear to have the potential to correctly identify patients with active epilepsy even in recordings with normal-appearing scalp EEGs, provided that their EEG-focus was determined in a previous work-up (e.g. with a sleep EEG). Patients with chronic focal epilepsy are particularly difficult to monitor with respect to drug response, given that they are often amnesic for their seizures, in particular if seizures involve the left-temporal lobe (Lux *et al.*, 2002). Studies with implanted devices found major discrepancies between reported and actually occurring seizures (Cook *et al.*, 2013). In contrast, in patients with generalized epilepsy, provocation manoeuvres, like hyperventilation or photic stimulation, or morning EEGs allow determining relatively easily with visual analysis if the epilepsy is controlled with reasonable certainty (Fittipaldi *et al.*, 2001; Labate *et al.*, 2007).

The results of this study are also in line with our previous EEG-functional magnetic resonance imaging findings which showed that presumably negative EEG from surgical candidates obtained inside the magnetic resonance imaging scanner contains spike-map that can be used to identify the epileptogenic focus reflected by corresponding regions of blood oxygenation level-dependent changes (Grouiller *et al.*, 2011). In 78% of the patients, the localization was correct using very stringent criteria, i.e., complete seizure control after resection of the so-identified area. In a more recent study, we could also show the existence of the patient’s individual epileptic network, detectable as haemodynamic map in the functional magnetic resonance imaging, which is largely preserved even when the contribution of spikes detected on scalp EEG is removed (Iannotti *et al.*, 2016).

Visible spike activity represents most likely only the ‘tip of the iceberg’ of the pathological focal epileptic activity (Alarcon *et al.*, 1994; Tao *et al.*, 2007; von Ellenrieder *et al.*, 2014). Spikes may remain spatially limited in deep structures and not recruit the necessary 4–6 cm^3^ of the cortex to be visible (Tao *et al.*, 2007). Nevertheless, their presence is strong and frequent enough to shape the scalp EEG using the whole-scalp topography even when they are not discernable using conventional visual inspection methods. Indeed, a study (Koessler *et al.*, 2015) on seven patients recorded with simultaneous scalp and intracranial EEG showed that deep cerebral sources (i.e. mesial temporal) are not visible on the scalp at the single sweep level, due to too low signal-to-noise ratio, but contribute to the topography in the scalp EEG. Our finding strengthens this assumption: when we averaged the EEG at the moment of maximal correlation of the spike-map in the no-spike-condition condition, we found no clearly visible abnormality in the EEG traces, neither low amplitude discharges nor focal slow waves, both in single sweeps and after averaging.

There is still controversy about the relationship between the appearance of IEDs and seizure risk. A review based on animal studies suggested a rather protective effect (De Curtis and Avanzini, 2001). In patients, it appears that a higher IED frequency correlates with a higher seizure likelihood. Baud *et al.* (2018) analysed data from intracranial recordings in 37 patients who received a closed-loop implantable brain stimulator, allowing hourly calculated IEDs and seizures over a median of 2.3 years. Seizures occurred preferentially during periods of high IED load, indicating that IEDs carry prognostic information on seizure probability. While the correlation is not absolute, it matches the clinical impression that seizure relapses in focal epilepsy are often associated with an increase of pathological discharges in the scalp EEG.

In two cases (8%), the spike-map was more prevalent in the EEG of healthy controls than in the no-spike-condition EEG of the patients. In both cases, the maps resembled strongly physiological microstates, i.e., maps that correlate with blood oxygenation level-dependent signal changes labelled as auditory (Patient 8) and visual resting-state networks (Patient 19, Fig. 5; Britz *et al.*, 2010). In fact, in the scalp EEG of healthy subjects, 4–11 resting states were identified, which can be also retrieved in resting functional magnetic resonance imaging (Britz *et al.*, 2010; Musso *et al.*, 2010).

In the current study, we were interested to determine if spike-related voltage maps are specific enough to be used as a marker of epileptic activity. The comparison with healthy control allowed determining if spike-maps were unique for patients or if they were also found in the EEGs of non-epileptic subjects. If spike-maps occurred equally often in both groups, they would be useless as a marker of epileptogenic activity because they would occur also as part of normal brain activity. In all but two subjects (92%), individual spike-maps were identified which were clearly different from physiological maps in the control group. Regarding patients with spike-maps mimicking physiological maps, future studies will show if more electrodes (e.g. 64 electrodes) allow better differentiation from controls.

The results indicate the presence of epileptogenic activity even if the EEG is unrevealing. While this has been shown with the help of functional magnetic resonance imaging or intracranial recordings, we now present evidence that hidden epileptogenic activity can be reliably recognized in scalp EEG. Spike-maps were more frequent but shorter than in controls. These findings corroborate clinical observations that epileptogenic activity is of frequent but short and sudden appearance leading to the well-described interictal cognitive deficits.

The number and type of antiepileptic drugs taken by our cohort of patients were not a contributing factor to our findings. However, we only examined patients with epilepsy who were candidates for epilepsy surgery. By definition, their epilepsy did not respond to at least two drugs. These patients have seizures under full drug regimen, i.e., chronic epileptogenicity can be readily assumed, with the moderate impact of partial drug withdrawal.

While we used only wake EEG, brief light sleep might have occurred increasing the likelihood of subtle or visible epileptogenic activity. However, in the no-spike-condition, there were no differences in the presence of spike-maps retrieved between EEGs containing mainly lower or higher frequencies.

It is of note that the presence of spike-maps was not correlated with clinical variables of severity such as duration of disease, presence of a structural lesion or history of generalized tonic-clonic seizures. With the inherent limitations of a small study, our results indicate that spike-maps are a robust marker of epileptogenic activity and relatively independent from disease evolution or vigilance state.

Studies with larger patient populations will help to establish a sensitivity, specificity, positive and negative predictive values of spike-maps as a marker of epileptogenic activity in patients with focal epilepsy. Cut-off values of good versus deficient epilepsy control or epileptic versus non-epileptic conditions will also need to be established. Further studies will help to determine if our findings extend also to other clinical scenarios, for example, the utility of spike-maps as a marker of long-lasting drug response after the first seizure or for compliance monitoring. Finally, the question of a universal pathological map or a repertoire of maps in patients as part of their initial work-up after a suspicious event is of major ongoing interest. While it may not be related to the presence of a particular spike-map, changes in the relative frequency or duration of physiological maps could provide a hint for the presence of a previously undetected epilepsy disorder.

## Funding

The study was supported by the Swiss National Science Foundation (169198, 163398, 192749, Sinergia CRS115-180365 and 170873) and Fondation Privé HUG No RC1-23.

## Competing interests

M.S. and S.V. have shares in Epilog; M.S. received speakers’ fees from EGI-Philipps and Desitin. The other authors report no disclosures.

## Abbreviations

CBZ =: carbamazepine
CLB =: clobazam
CNZ =: clonazepam
DNET=: dysembryoplastic neuroepithelial tumour
FCD =: focal cortical dysplasia
GEV =: global explained variance
GFP =: global field power
HS =: hippocampus sclerosis
IEDs =: interictal epileptiform discharges
LC =: left-central
LCP =: left-central-parietal
LCS =: lacosamide
LEV =: levetiracetam
LF =: left-frontal
LPO =: left-parietal-occipital
LT =: left-temporal
LTG =: lamotrigine
OXC =: oxcarbazepine
PNES =: psychogenic non-epileptic epileptic seizure
RF =: right-frontal
RFN =: rufinamide
ROF =: right-occipital-frontal
RT =: right-temporal
sGEV =: standardized global explained variance
TPM =: topiramate
VPA =: valproate
ZNS =: zonisamide
